# Shape Self-Regulation in Early Lung Morphogenesis

**DOI:** 10.1371/journal.pone.0036925

**Published:** 2012-05-16

**Authors:** Raphaël Clément, Pierre Blanc, Benjamin Mauroy, Vincent Sapin, Stéphane Douady

**Affiliations:** 1 Laboratoire Matière & Systèmes Complexes, UMR CNRS 7057, University Paris Diderot, Paris, France; 2 Laboratoire J. A. Dieudonné, UMR CNRS 7531, University Nice Sophia-Antipolis, Nice, France; 3 EA R2D2, Medicine School, Auvergne University, Clermont-Ferrand, France; Childrens Hospital Los Angeles, United States of America

## Abstract

The arborescent architecture of mammalian conductive airways results from the repeated branching of lung endoderm into surrounding mesoderm. Subsequent lung’s striking geometrical features have long raised the question of developmental mechanisms involved in morphogenesis. Many molecular actors have been identified, and several studies demonstrated the central role of *Fgf10* and *Shh* in growth and branching. However, the actual branching mechanism and the way branching events are organized at the organ scale to achieve a self-avoiding tree remain to be understood through a model compatible with evidenced signaling. In this paper we show that the mere diffusion of FGF10 from distal mesenchyme involves differential epithelial proliferation that spontaneously leads to branching. Modeling FGF10 diffusion from sub-mesothelial mesenchyme where *Fgf10* is known to be expressed and computing epithelial and mesenchymal growth in a coupled manner, we found that the resulting laplacian dynamics precisely accounts for the patterning of FGF10-induced genes, and that it spontaneously involves differential proliferation leading to a self-avoiding and space-filling tree, through mechanisms that we detail. The tree’s fine morphological features depend on the epithelial growth response to FGF10, underlain by the lung’s complex regulatory network. Notably, our results suggest that no branching information has to be encoded and that no master routine is required to organize branching events at the organ scale. Despite its simplicity, this model identifies key mechanisms of lung development, from branching to organ-scale organization, and could prove relevant to the development of other branched organs relying on similar pathways.

## Introduction

Regulation of early lung development has been the subject of intensive research over the past few decades. The main issue is to understand how elementary branching events occur, in other words how an epithelial tube undergoes tip-splitting, and how these branching events are organized throughout development to achieve a self-avoiding bronchial tree [1], [2]. Bronchi indeed never meet one another nor reach the pleural mesothelium enclosing the mesenchyme, which introduces a typical distance from distal buds to mesothelium. These aspects of lung geometry are rarely considered in relevant literature or in developmental models [1], [3], [4], although they are highly non-trivial in this confined geometry. Such striking features should be accounted for in any attempt to model lung development, as they must somehow witness the mechanisms involved in branching.

Experimental research provided crucial information concerning the molecular aspects of shape regulation, and several works contributed to evidence the main actors involved. Among others, the central role of *Fgf10* has been demonstrated: it has been reported to be responsible for epithelial proliferation [5], [6], and null mutants of *Fgf10* or of its receptor *Fgfr2b* have been reported to present lung agenesis [7], [8]. SHH have been shown to down-regulate *Fgf10* expression in the proximal mesenchyme [6], [9]. *Fgf10* expression is consequently restricted to the distal mesenchyme [10]. Also, *Shh* as well as *Spry2,* which inhibits FGF10-induced epithelial proliferation, are expressed at high levels in distal epithelial cells [10], [11] but at very low levels between buds. Further analysis shows that *Spry2* expression is up-regulated by FGF10 reception by epithelial FGFR2b, suggesting that FGF10 income concentrates on epithelial buds. These insights into molecular regulation of lung development have led to several genetic models [2], [3]. However, no actual branching mechanism has been explicitly described, although it has been proposed that *Fgf10* split-expression could prefigure future branching events, or that a fibronectin deposit could lead to bud tip-splitting [3], [6]. Similarly, no explicit mechanism has been proposed to account for the organ-scale organization.

More recently, it has been suggested that the tremendous amount of encoding apparently needed to organize the bronchial tree could be considerably reduced [4]: authors propose that epithelial bud branching could rely on a “programme” involving three elementary modes of branching combined in three sequences. Successive branching events would result from the exhaustively encoded action of hypothetic operators, leading to a stereotyped bronchial tree. But a self-avoiding structure might then not be achieved unless the size and direction of each bud are rigorously specified. However, regularly observed “errors” in branching lineages do not lead to bud collision, as one would expect in this scenario. Also, if first generations seem indeed stereotyped, statistical analysis of morphometric data suggests that following generations rather adapt to fill the mesenchymal volume than follow a stereotyped routine [12].

Mathematical growth models have also been introduced, but very few proposed explicit mechanisms for branching or organization. Notably, Lubkin and Murray published in 1995 a model based on viscous fingering, highlighting a spontaneous branching mechanism, but with no role for signaling molecules now known to be required [13]. More recently, Miura and Shiota proposed a reaction-diffusion model, this time based on the role of signaling molecules. Spontaneous branching is very interestingly observed, but the study is restricted to radial geometry and to in vitro development of epithelial cells exposed to FGF1 [14]. In vivo, *Fgf10* patterning plays a crucial role as its homogeneous expression in *Shh* null mutants leads to “sac growth” and branching failure [9]. Also focusing on signaling molecules, Hirashima et al. modeled *Fgf10* patterning together with the diffusion of relevant proteins, showing that split-expression may emerge in the reaction-diffusion process. However the model does not implement growth, so the link between patterning and shape remains missing [15]. A very recent attempt has been similarly made to integrate the core signaling network into a reaction-diffusion formalism [16]. However, the model is restricted to the description of concentrations and expression patterns. Although authors claim that obtained concentration patterns prefigure branching events, this remains questionable, as growth is still not implemented with regards to the presence of signaling molecules, and as predicted expression patterns are very different from those observed in vivo.

Existing models for lung development finally seem either to lack basic mechanisms accounting for the striking emerging features of the shape: branching, self-avoiding organization, and epithelium to mesothelium distance; or not to account for evidenced molecular regulation. In this paper we will introduce an organ-scale model based on reported experimental evidences, where the motion of both epithelium and mesothelium are implemented, and where epithelial growth is a function of FGF10 reception after diffusion in the mesenchyme. We will see that *Fgf10* distal patterning leads to differential epithelial proliferation, involving spontaneous bud branching as well as self-organization of the tree, and we will describe in details the mechanisms involved.

## Methods

### Whole-mount in Situ Hybridization

Animal procedures followed the French and European guidelines with the approval of the local ethics committee of animal care and use (CEMEA Auvergne # B63-175). All mice were maintained in plastic cages with ad libitum access to food and water. Mouse embryos (CD1) were dissected in cold (4°C) PBS and lungs were immediately fixed in 4% paraformaldehyde/PBS (wt/vol) at 4°C, with gentle rocking for 1 hour. Fixed lungs were washed in PBS for 5 min at room temperature, with gentle rocking. Lungs were then dehydrated by washing once in 25% methanol/PBT (PBS with 0.1% (vol/vol) Tween-20), once in 50% methanol/PBT, once in 75% methanol/PBT, and twice in 100% methanol. Dehydrated specimens were stored at −20°C in 100% methanol (some of them for 10 months before use without obvious deterioration of staining results). Following steps were carried out into 2 ml RNAse Free tubes and in 1 ml of reagent, at room temperature with gentle rocking, unless otherwise stated. On day 1, dehydrated lungs where rehydrated through an inverted methanol/PBT series (5 min washes in each of 75%, 50% and 25% methanol/PBT, followed by 2×5 min washes in PBT). Lungs were permeabilized 5–6 min (depending on the size of the lungs) with 10 µg/ml proteinase k/PBT and digestion was stopped by washing 5 min with 2 mg/ml glycine/PBT. Specimens were washed 2×5 min in PBT, refixed for 20 min in 0.2% glutaraldéhyde/4% paraformaldehyde, and washed again 2×5 min in PBT. Lungs were then incubated in hybridization solution (50% formamide (vol/vol), 5×SSC, 1% SDS, 0.1 mg/ml of yeast RNA, 0.05 mg/ml of heparine) for 1 h at 65°C, and then they were incubated overnight at 65°C in a 1 µg UTP-DIG labeled probe/300 µl hybridization solution. The cDNA used as template for the Fgf10 riboprobes (pKS-mFgf10) was provided by Dr. S. Bellusci. On day 2, lungs were rinsed twice (10 min and 30 min) at 65°C with washing solution n°1 (50% formamide (vol/vol), 5×SCC, 1% SDS), 30 min at 65°C with washing solution n°2 (50% formamide (vol/vol), 2×SCC) and 2×5 min with TNT (100 mM Tris pH 7.5/150 mM NaCl/0.1% Tween-20). Blocking was performed trough 1 hour wash in TNB (0.5% (wt/vol) Blocking Reagent (Perkin Elmer/TNT). Lungs were incubated 2 hours with the Antidigoxigenin-AP Fab fragments (Roche) diluted 1∶1000 in TNB. A series of washes with TNT was carried out (5 min - 10 min –15 min and 3×20 min). Lungs were then washed 3×5 min in NTMT (100 mM Tris pH 9.5/100 mM NaCl/50 mM MgCl2/0.1% Tween-20). Specimens were incubated overnight with a 20 µl NBT-BCIP (Roche)/1 ml NTMT solution. Staining reaction was stopped by removing the mix above and adding 1 ml of cold (4°) PBS. Finally lungs were transferred in 4-well plates (Nucleon Surface NUNC) and imaged using SZX12 Olympus.

### 3D Reconstructions of Mouse Fetal Lungs

Mouse Embryos were collected at embryonic day and dissected in cold (4°) PBS (head and caudal end below the liver were removed). For histological analysis, dissected trunks were fixed in AFA (alcohol+formalin+acetic acid) for 24 h at room temperature, embedded in paraffin, cut at 5 µm and stained with HPS (hematoxylin-phloxine-saffron). The HPS stained section series were arrayed on microscope slides, snapshots were taken at low magnification (x4) and images were stacked using MetaMorph. Image stacks were aligned using FIJI. 3D reconstructions of the full cranial lobes were performed on Bitplane Imaris. For relevant calculations, meshes of the epithelial and mesothelial reconstructed surfaces were exported to Comsol Multiphysics in which Laplace’s equation was solved using the finite elements method.

**Figure 1:**
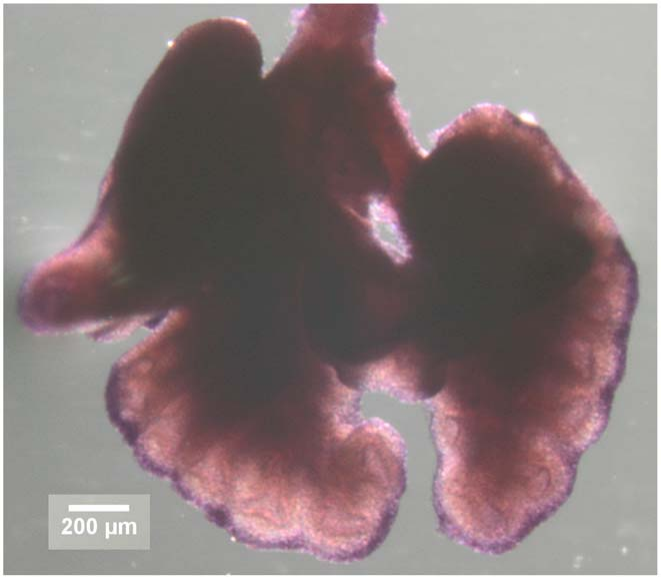
Fgf10 patterning at E13.5. Ventral and dorsal views of mouse lung at embryonic day E13.5. Whole mount in situ hybridization shows that *Fgf10* expression is strongly restricted to the distal part of the mesenchyme.

### Construction of the Model

Building a formal description of lung development requires starting from basic lung geometry and modeling FGF10 diffusion and reception by epithelial cells. We thus consider two moving surfaces, the epithelium and the mesothelium. The concentration *c* of FGF10 in the mesenchyme obeys to the general law of diffusion:
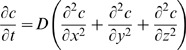
(1)where D is FGF10 diffusion coefficient in the mesenchyme. As we can consider that growth is slower than diffusion, FGF10 concentration is at equilibrium and simply obeys Laplace’s equation:
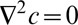
(2)with boundary conditions: *c_min_* (epithelium) and *c_max_* (mesothelium).

**Figure 2:**
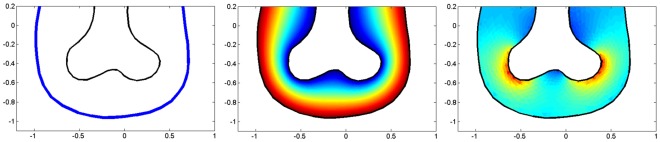
Construction of the model. (**A**) Naive branching geometry. The epithelium (black) is undergoing branching. *Fgf10* expression is restricted to the sub-mesothelial mesenchyme. In the model, only the most distal cells are source of FGF10 (blue). (**B**) Resolution of Laplace’s equation for FGF10 concentration *c* in the same geometry using finite elements methods. Near the mesothelium the concentration is maximum (red), while it is minimum on the epithelium (blue). (**C**) Calculation of the gradient norm of FGF10 concentration in the same geometry. Blue stands for weak gradients while red stands for high gradients. Gradient focuses on distal tips.

This equation can be solved numerically if boundary conditions are specified. As quantitative data on FGF10 concentration is unavailable, it seems more relevant to start from the expression pattern, which is very well known. As it was previously reported, whole-mount in situ hybridizations in mouse lung, even here at embryonic day E13.5, suggest that *Fgf10* expression is restricted to the most distal cells of the mesenchyme (Fig. 1). The fact that *Fgf10* patterning remains distal never mind the moment considered shows that it is not very sensitive to the deformation of the expression domain. This is also consistent with the equilibrium hypothesis, which assumes slow deformation of the tissues. Therefore we will use as first boundary condition that the concentration of FGF10 is maximal (*c_max_*) near the mesothelium, from where it diffuses. As *Fgf10* distal patterning relies on SHH, it implicitly includes the role of SHH. The second boundary condition is underlain by FGF10 reception by epithelial cells. As FGF10 binds to epithelial FGFR2b, its degradation induces that FGF10 concentration is minimum (*c_min_*) on the epithelium. *c_min_* and *c_max_* are set to 0 and 1 respectively. Given these boundary conditions, we can solve Eq. 2 in any geometry to compute a model field of FGF10 concentration. A demonstrative calculation is presented Fig.2, where FGF10 expression, concentration and gradient are displayed in a naive branching geometry.

The concentration jump from epithelium to mesothelium, biologically underlain by *Fgf10* patterning and by FGF10 consumption, causes diffusion of FGF10 along the gradients. This means that diffusion tends to spatially equilibrate concentrations, idea formalized by Fick’s law of diffusion: the diffusive flux of a molecular species is proportional to the gradient of its concentration. It is then easy to obtain the diffusive flux of FGF10 in the mesenchyme in the model: it is simply proportional to the gradient of concentration. The numerical solution of Laplace’s equation is calculated using the finite elements method. The interfacial resolution of the mesh introduces a cut-off length that stands for surface tension: physically, surface tension underlies the energetic cost associated to new surface creation, i.e. to curvature. For any free surface, such as the epithelial sheet, it involves a persistence length related to the rigidity of the surface, and thus to the strength of cell-cell bonds. In the growth model, we chose not to introduce the equations of mechanics: as found later, the branching mechanism uncovered is more general than its mechanical context and a complete formalization of the mechanics is not required to understand the branching process. We thus use the cut-off length as an ad-hoc physical input to account for the rigidity of the surfaces. Physically, spatial perturbations under the persistence length are smoothed; in the model, the cut-off has the same role.

### Growth Simulations

The simulations are computed with Matlab and Matlab Partial Differential Equation Toolbox, which is used to solve Laplace’s equation on the deforming geometry with the finite elements method. The initial condition is set to a tubular geometry, with lengths in arbitrary units: outer radius 1 (mesothelium), inner radius 0.2 (epithelium) and height 10. The time increment is set to 0.05. Details of the algorithm structure and of parameters values are given in supplementary material (Figs. S1, S2).

## Results

### FGF10 Diffusion Accounts for Spry2 Patterning

**Figure 3:**
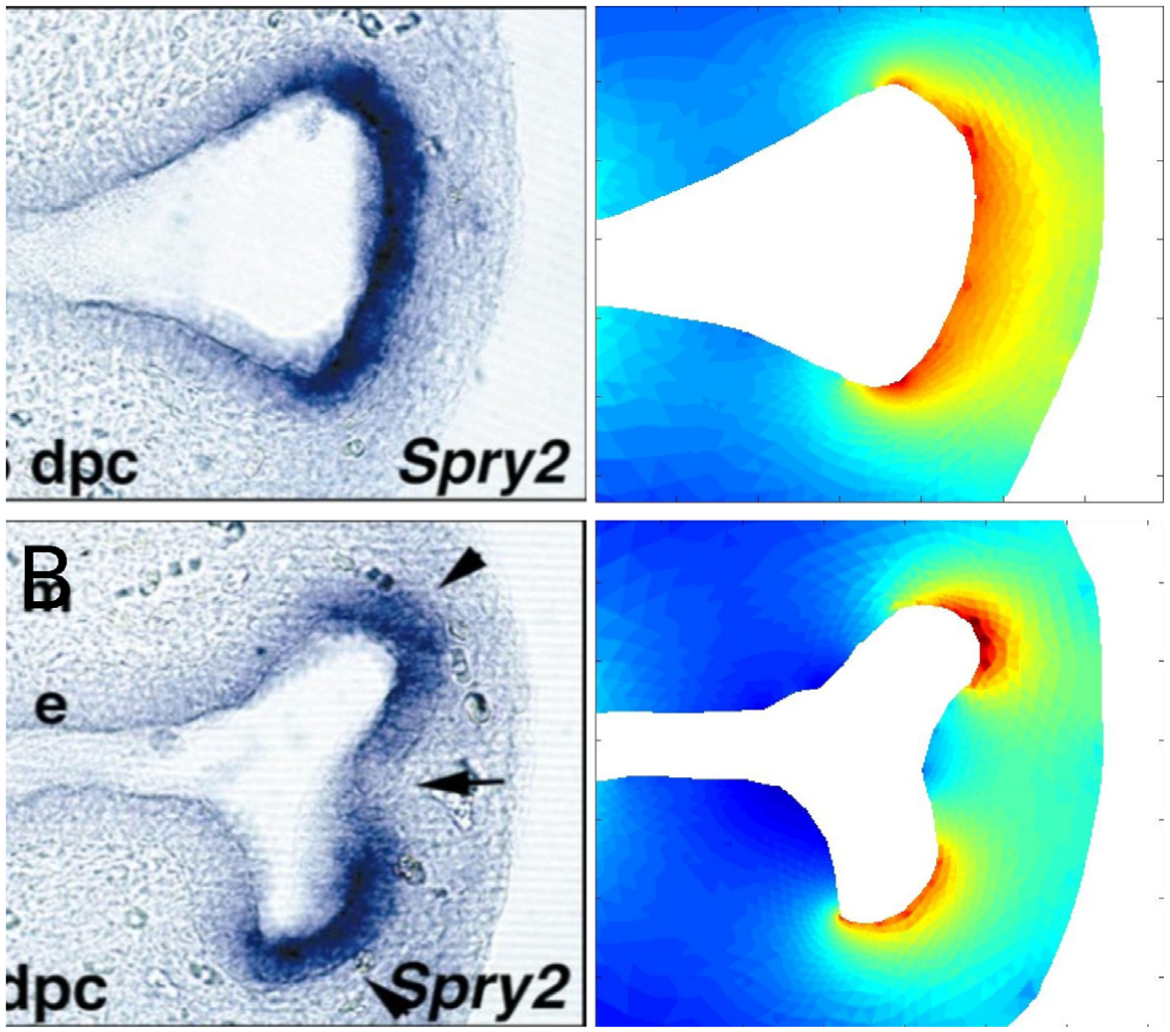
FGF10 diffusion accounts for Spry2 patterning. (**A**) *Spry2* whole mount in situ hybridizations before and after a branching event, courtesy of S. Bellusci [11]. Before the branching event, *Spry2* expression spreads on the entire bud’s width. After branching has occurred, *Spry2* expression splits and focuses on each new bud, while it weakens at the branching point. As *Spry2* expression is induced by FGF10, *Spry2* reports FGF10 reception. (**B**) Calculation of FGF10 flux predicted by the model in the same geometry. No adjustable parameters are used. Before the branching event, FGF10 diffusive flux spreads on the entire bud’s width. After branching has occurred, FGF10 flux spontaneously focuses on distal tips, just as *Spry2* expression. Although it only considers FGF10 diffusion from distal mesenchyme, the model accounts for the patterning of *Spry2*.

**Figure 4:**
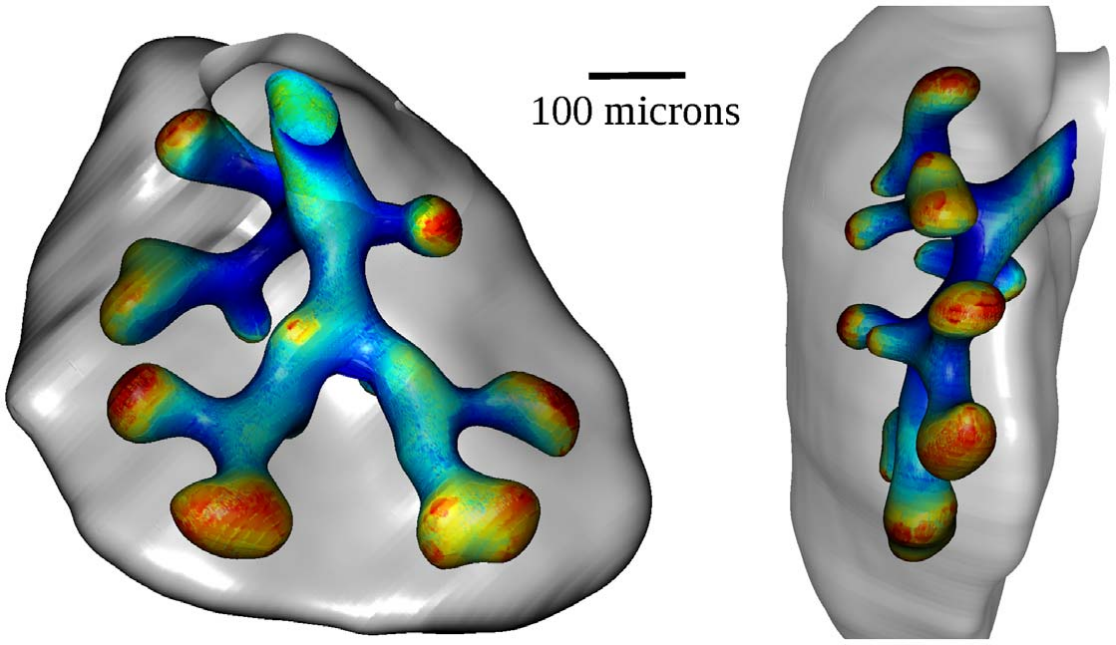
Gradient calculation in 3D reconstructions. Calculation of FGF10 flux in a 3D geometry reconstructed from embryonic mouse right cranial lobe at E12.5 with the same laplacian model and boundary conditions. The left image presents an upper view while the right image presents a side view. Both epithelial and mesothelial surfaces are displayed. The color code on the epithelial surface stands for the received flux. The same tip-effect is found in this geometry.

The model for FGF10 concentration and flux can be confronted to experimental imaging. No imaging of FGF10 concentration during lung development is available, however literature reports that *Spry2* expression by epithelial cells is induced by FGF10 reception. *Spry2* is thus an indirect reporter of FGF10 income on the epithelium. We mapped, in the same geometry, the model flux of FGF10 compared to *Spry2* whole mount in situ hybridization (courtesy of S. Bellusci). As shows Fig. 3, the agreement between the model and the experiment is excellent in this case. Notably, it validates the equilibrium approximation that led to Eq.2. There are no adjustable parameters since we directly solved Eq. 2. Both *Spry2* expression and FGF10 flux are focused on distal tips of the buds, and are very sensitive to local geometry, as shows the post-branching image (Fig. 3B) where both expression and flux are very low at the branching point. To confirm that this behavior is general and also found for lung 3D geometry, we calculated the model flux of FGF10 predicted by the model in a 3D reconstruction of mouse right cranial lobe. The same effect is observed: flux is spontaneously higher on distal tips (Fig. 4). This can in fact be interpreted as a geometrical effect of FGF10 diffusion, since the resulting laplacian field and its gradient are dramatically sensitive to local distance to mesothelium and to local epithelium curvature. This screening effect or tip-effect for the gradient, here for the flux of FGF10, has been described in several other laplacian systems [17], [18], and is for instance responsible for the efficiency of lightning rods that concentrate the electromagnetic field. These results show that the mere diffusion of FGF10 from distal mesenchyme during lung morphogenesis accounts for both the non-trivial patterning of FGF10-induced genes such as *Spry2* and for the tip-localized growth of lung epithelium.

Also, the results point out the sensitivity to the flux of FGF10, both in the simple branching geometry and in the reconstructed lobe. It is often assumed that cells are rather sensitive to concentration than to flux. However, sensitivity requires measuring an amount of interactions or shocks per unit of time, and such objects are underlain by the flux. Unless it moves quickly in the medium, a receptor or detector cannot count surrounding particles, but needs to count how many particles reached it through diffusion. Here FGF10 binds to FGFR2b only if the first “hits” the second, and the amount of FGF10 received by epithelial cells is consequently related to the flux. It is thus no surprise that the relevant object to consider here is the gradient of concentration. It has been reported that exogenous FGF10 addition in the mesenchyme triggers *Spry2* expression [11]. At first glance, this result could shed doubt on a flux-based sensitivity, as FGF10 concentration is initially more or less homogeneous. But the binding of FGF10 to epithelial FGFR2b in fact quickly restores a concentration gradient, and thus a gradient-oriented diffusive flux, bringing the system back to the assumed steady state.

### FGF10 Diffusion Accounts for Branching Morphogenesis

**Figure 5:**
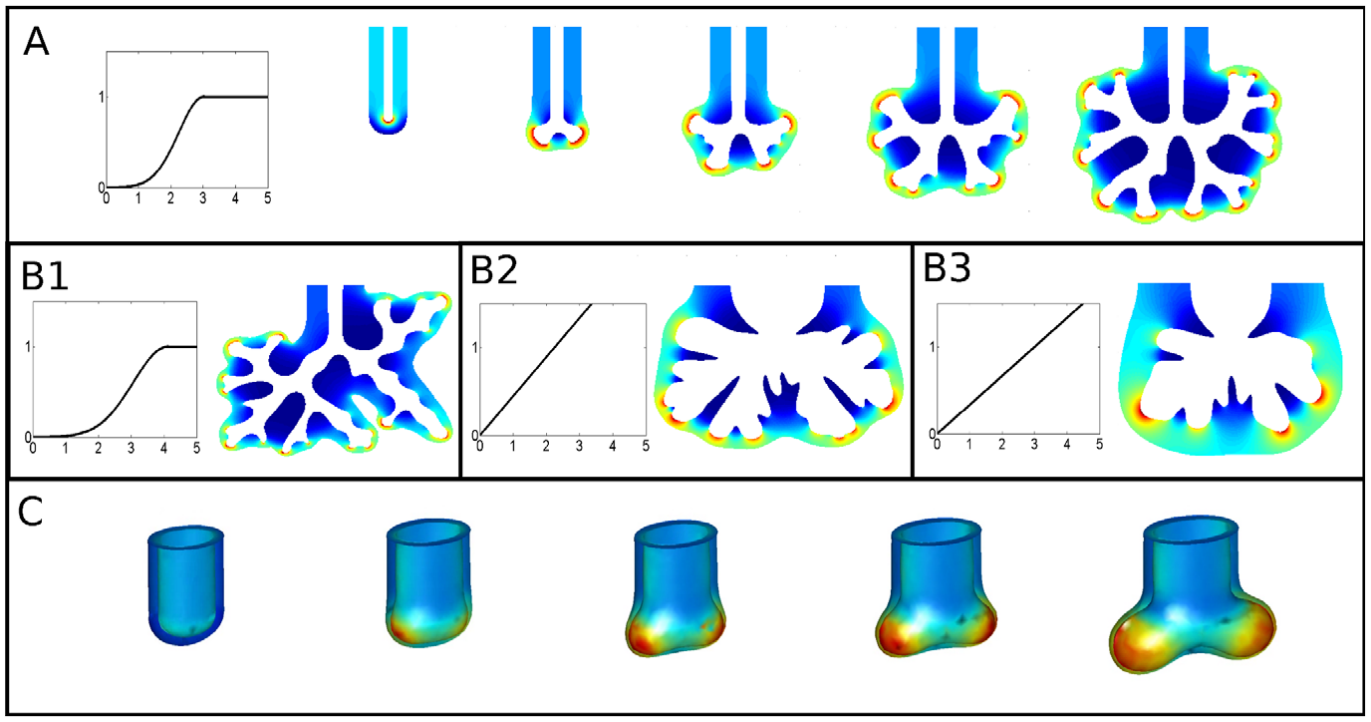
FGF10 diffusion accounts for branching morphogenesis. (**A**) Time lapse sequence of a growth simulation. The simulation relies on the laplacian model and couples the motion of the epithelial and mesothelial surfaces. Left image is a plot of the sigmoid growth response to FGF10 corresponding to the simulation. Colors in the mesenchyme stand for FGF10 flux, which focuses on distal tips. Branching occurs spontaneously and branches never meet each other, as observed in vivo. The epithelium to mesothelium distance is conserved, as branches never reach the mesothelium. (**B**) Results of three simulations with their respective growth responses. B1 features an other sigmoid growth response with a different spread. B2 and B3 feature linear growth responses with different values of *g*. The initial condition is always the initial tube displayed in A. The arbitrary scale is chosen the same for all (A) and (B) simulation results. While the morphologies obtained vary with the growth response, the initial non-branched tube always develops into a self-avoiding tree. (**C**) Time lapse sequence of a bud-scale 3D simulation based on the same model. The initial tube branches, while FGF10 flux focuses at bud tips, showing that the model and mechanisms are relevant to 3D geometry.

The excellent agreement found Fig. 3 shows that the model succeeds to reproduce the behavior of FGF10 dynamics in the mesenchyme and shows the importance of this geometrical “tip-effect” in a branched structure. To test if this effect can account for the formation of buds in the first place, we computed growth simulations based on the same model. The initial tubular geometry is chosen close to a bronchial tube before any branching event has occurred. In this geometry we calculate the concentration of FGF10 in the mesenchyme and its diffusive flux. Although the epithelium-mesothelium distance is chosen constant for the initial condition, it is worth noticing the critical effect of curvature, which already implies a higher FGF10 diffusive flux at epithelial tip (Fig. 5 - initial condition).

The local normal velocity of the epithelium (*u_e_*) is written in a very general manner as a function of locally received FGF10, therefore to the local gradient, as discussed before. In this model we will simply consider that FGF10 reception directly induces normal surface growth. Cell proliferation, even tangential as it has been reported to be during bud growth [19], can indeed only lead to normal motion of the surface. The direction of this normal motion (outwards) is explained by both lumen’s higher pressure and possible pre-existing epithelial curvature. The local transformation of tangential proliferation into normal motion in fact relies on the mechanical properties and rigidity of the tissues. As we discussed in the methods section, our model does not consider mechanics but rather an ad-hoc numerical persistence length. As we will see, this numerical length indeed plays its mechanical role. The local motion of the mesothelium (*u_m_*) has two contributing terms. The first one accounts for the motion of the epithelium: the mesenchyme is incompressible and transmits the motion to the mesothelium 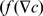. The second term accounts for mesenchymal proliferation. Proliferation of mesenchymal cells has been reported to be regulated by the SHH/FGF9 pathways [20]. SHH is produced on the distal epithelium [10] and diffuses from the tips, where FGF10 is received. Mesenchymal growth should thus be more important in zones of strong epithelial proliferation (distal tips). Therefore we also take the second term proportional to 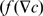
with an a priori unknown prefactor *g*. This formulation keeps the model as simple and general as possible, but still integrates subtleties of mesenchymal proliferation, which is required to model the motion of epithelium and mesothelium in a coupled manner. As FGF10 income on epithelial cells is proportional to the gradient of concentration, growth finally reads as follows (see Fig. S2 for details) :


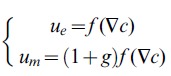
(3)

The growth response *f* theoretically involves the biology underlying FGF10-induced growth as well as the physics of the epithelium-mesenchyme interface. We a priori have very little quantitative information about this growth response. However, the following qualitative behavior can be proposed: as FGF10 contributes to epithelial proliferation, we assume that the growth response increases with the flux. It is likely that for very low FGF10 income, no or very little proliferation is induced. Also, intra-cellular down-regulation of the FGF10 pathway by *Spry2* may involve saturation or even decrease of the proliferation for too high values of the flux. The most likely is that the growth response follows a smooth variation from no growth to a maximal proliferation rate, which is very well represented by a sigmoid *f* function.

Results of growth simulations are presented in Fig. 5. At each iteration of a simulation, FGF10 concentration is first calculated according to Eq. 2, and then the local motion of the boundaries is computed according to Eq. 3 (see Fig. S1). Fig. 5A shows a time lapse sequence of a simulation obtained using a sigmoid growth response (see corresponding movie S1). The resulting shape does present great analogies with lung morphology. The initial bronchial tube spontaneously undergoes repeated branching while the mesothelium remains at fixed distance of distal tips (see Figure S4), just as observed in vivo. Branching appears to be either tip-splitting or side-branching (Figs. 5A, 5B1), as reported in literature [4]. One also observes three-way branching events (Fig. 5B1), which is commonly found in vivo in available imaging. Similarly to what has been reported and to our previous findings (Fig. 3–4), proliferation is inhomogeneous and focuses on bud tips. Finally, tree is space-filling and self-avoiding; in a spontaneous manner since no organization routine of any kind is implemented in the simulations. Fig. 5B shows the outcome of a few simulations featuring various growth responses. The main point is to show that the previous results are very robust since all the striking features of lung geometry are still found when the growth response is modified (Fig. S6). Even in the rough case of a linear relationship between flux and growth, the branching structure is preserved. However, the type of growth response and the relative growth of the mesenchyme do have an influence on “second order” geometrical properties of the tree, such as branches width or branching regularity. The formulation of the model allows being more precise: it is possible to systematically test the effect of modifications in the growth response. Additional data for instance suggests that a low sensibility to small fluxes favors elongation rather than lateral growth, which seems logical since branches sides receive small fluxes. The relative growth of the mesenchyme can also be tested, and results suggest that the weaker it is, the denser is the resulting tree, as one could have expected. On the contrary, higher mesenchymal proliferation leads to looser trees (Fig. S3). Last, we chose to work in 2D to keep computation times reasonable. However, a few runs performed in 3D confirmed that a similar behavior is obtained and that the model is also fully relevant to 3D geometry (Fig. 5C).

## Discussion

### Branching Mechanism and Organ-scale Self-organization

**Figure 6:**
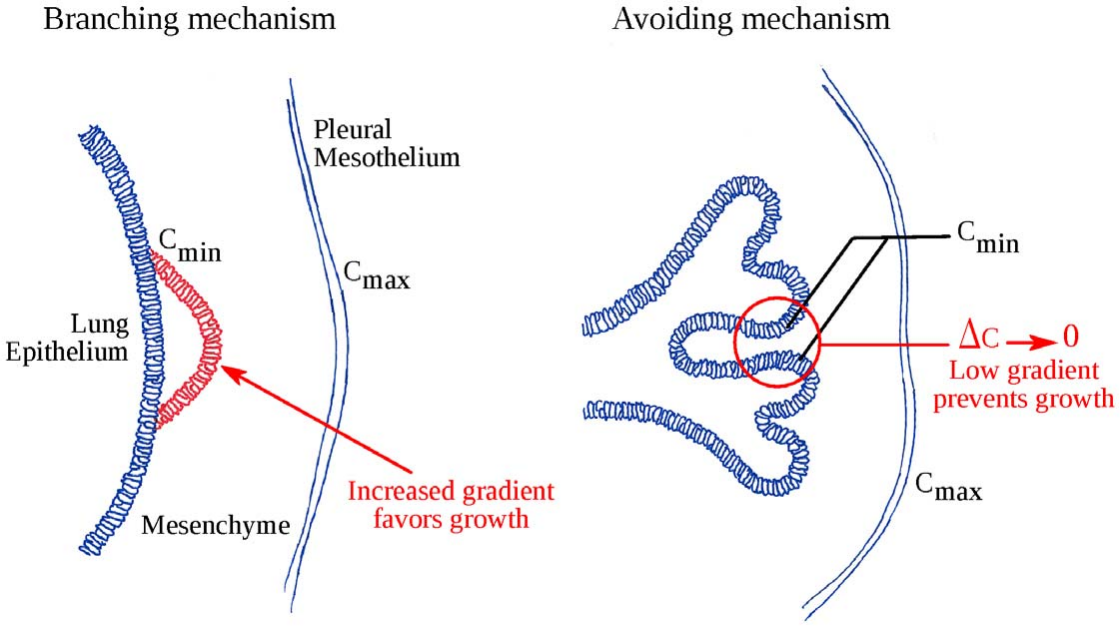
Branching mechanism and organ-scale self-organization. (**A**) The branching mechanism. Consider any prominence on the epithelium (here displayed in red). This bud increases the local gradient of concentration and thus the local flux of FGF10 received by epithelial cells. It will thus grow faster and be amplified. This instability mechanism is balanced by surface tension, which prevents thin branches to form. (**B**) The mechanism of self-organization. When two branches get too close, the local gradient of concentration in the interstitial mesenchyme tends to zero (red circle). Growth thus tends to zero and prevents branches from any collision. This mechanism, at the organ scale, leads to the self-avoiding bronchial tree.

Our results suggest that simple diffusion accounts for the expression of FGF10-induced genes, and then that it implies differential growth leading to branching. This spontaneous differential growth has a simple geometrical origin: Let us consider a small bud on a homogeneously growing epithelium. This bud, as small as it is, will locally increase the gradient and thus the flux of FGF10, because it is more curved and closer to mesothelium. Its cells will receive more FGF10 than the neighboring cells and its growth will be spontaneously favored compared to the surrounding epithelium. Without surface tension, the epithelium would be perfectly unstable and form infinitely thin branches. For the epithelial sheet, the effective persistence length relies on the mechanical properties of the epithelium and of the mesenchyme. As it introduces an energetic cost for curvature, it plays a stabilizing role and spatial instabilities of the epithelial surface are smoothed under a typical length-scale. Here the numerical cut-off plays the role of the mechanic persistence length (see Fig. S5 for more precise data concerning this cut-off), and as expected, controls the typical size of epithelial buckling. The epithelium becomes unstable beyond this length-scale, and undergoes branching when buds spread. We propose that this tip-effect on FGF10 gradient is the mechanism of lung epithelial branching during development (Fig. 6A).

The self-avoiding property of the tree is a geometrical effect inherent to this very mechanism. When two branches get too close to each other, the gradient and thus the local income of FGF10 tend to zero (Fig. 6B), preventing the tree from any bud collision. Other laplacian systems exhibit similar behavior in nature, from bacteria growth [21] to viscous fingering [22]. During lung morphogenesis, the epithelium branches in an enclosed growing media, and the dynamics is thus changed: in viscous fingering or bacteria growth, branches eventually reach the external boundary. Here it does not happen since a critical equilibrium distance appears, like it does in vivo (see for instance Fig. 4): bud tips shape and curvature spontaneously adapt according to *g* so that the values of the gradient on both epithelium and mesothelium allow that *u_e_ ≈ u_m_*. It is worth noticing that this equilibrium distance logically tends to zero when *g* tends to zero (see Fig. S3).

### Consistency of the Model

It is very important to check that a biological model is consistent with available mutant data, and to discuss its consistency with regards to phenotypes associated with gene defects. First, *Fgf10* null mutants display lung agenesis. This is trivially consistent with the model, in which epithelial proliferation is a function of FGF10 income. Second, *Shh* null mutants display no branching or severely impaired branching [9], and the ectopic expression of *Fgf10* in the whole mesenchyme [10]. Our model shows that the branching mechanism indeed requires *Fgf10* distal expression. Homogeneous expression of *Fgf10* would reduce the gradients and the tip effect, thus impairing branching, which relies on this tip effect. Note that homogeneous expression is quite different from an initially homogeneous concentration such as the one achieved in the experiment described in [11]: this last one still ends up with gradients as FGF10 binds to epithelial FGFR2b, and branching is conserved. Several other knock-outs have been performed, listed by Cardoso and Lu in their review. None of them seems to involve major shape changes. In other words, the striking features of the bronchial tree are not lost, but minor shape changes occur, such as the regularity of branching events or the size and shape of branches. This is qualitatively consistent with the model, which predicts minor shape changes when the growth response is modified. As they modify the regulation processes underlying the growth response, gene defects do modify the growth response itself. We want to point out that what we call here minor shape changes can involve major respiratory dysfunctions: they are only minor in terms of geometry.

The model is also consistent with observed branching asymmetry. Morphometric studies in mature lungs showed that branching events usually result in two branches of different sizes [23], [24], and available imaging at developmental stages confirms that this is already the case when branches form. Asymmetry exists in our model since branching results from a growth instability, which implies spontaneous symmetry breaking, whatever the dimension considered (2 or 3). Further studies and measurements of mean asymmetry ratios in the model and in vivo could prove very interesting, as asymmetry has been reported to play an important role in lung efficiency [25], [26].

The generality of the model has several counterparts. First, it cannot predict quantitatively the branching geometry of lung. Higher accuracy could be achieved if the growth response was better documented. For instance, one can imagine building a more realistic growth response experimentally, tracking the epithelial and mesothelial surfaces in vivo for a given species. This could be done comparing observed local growth to model-predicted flux. Also, the degradation of FGF10 signal is not considered. Although it would induce quantitative changes in the results, the qualitative mechanism of tip-effect would not be altered, as degradation would only modify the slope of the concentration spatial decay between the boundaries. Second, the stereotypy observed in first generations apparently finds no explanation here. This is not exactly the case since other laplacian growth instabilities have been reported to become very stereotyped when the external boundary is under a certain geometrical constraint [27]. After a few generations, instability takes over and branches fill available space. Lung morphogenesis is certainly not an isolated event in development, and constraints are applied by neighboring organs and tissues. It would be very interesting to see how the actual presence of other developing organs affects the first branching events stereotypy. Third, an explicit description of epithelial mechanics may prove useful. Both experimental measurements and theoretic work could shed light on the switch from tangential proliferation to normal motion, which somehow underlies the model. Last, this model would benefit from general 3D implementation of the simulations, as lung obviously does not grow in two dimensions. However, our results suggest that the mechanisms described are fully relevant to 3D geometry, both for the patterning of FGF10-induced genes and for the growth model.

### Conclusion

We first want to point out that this model, for several reasons listed earlier, has no ambition to quantitatively fit lung morphogenesis or to exhaustively account for the role of all the actors involved. Its main purpose was to qualitatively uncover the mechanisms inducing branching, self-avoiding, and other fundamental geometric features of embryonic lung, and to provide a framework for future studies. In other words, to determine which ingredients are actually needed and which are of “second order” for the emergence of shape’s striking features. We think that it does provide crucial insights into the actual branching mechanism, and a coherent scenario for early lung development that previously lacked. It shows that *Fgf10* does not carry any branching information, and that its “split-expression” is not required for branching, but that its distal expression is. Also, no master routine is required to spatially organize branching events. Cardoso & Lu pointed out that: “temporo-spatial restriction of *Fgf10* expression by SHH appears to be essential to initiate and maintain branching of lung” [2]. The mechanism we describe here establishes a direct link between spatially restricted expression and shape emergence. Quantitative modeling showed that *Fgf10* patterning could result from the diffusion processes of the FGF10/SHH/TGF-β regulation loop [15], although other regulation pathways, such as hydrostatic pressure, have been investigated [28]. Our work shows that with this distal patterning achieved, self-avoiding branching morphogenesis occurs spontaneously. The robustness of the global shape opposed to the plasticity of its fine geometric features have interesting consequences: as the growth response finely relies on lung’s regulatory network, the model provides an elegant framework to understand how the bronchial tree may have acquired its near-optimal geometry [29] through natural selection. The self-regulation of the shape (shape – patterning – growth – shape) constitutes a very economic way to achieve morphogenesis, and is likely to have been used in evolution, as it demands far less encoding than if every single branching event had to be specified individually. Searching for common molecular mechanisms in the morphogenesis of branched organs, Horowitz et al. pointed out that their prominent common feature was the duality of the pathways involved: an agonist and its inhibitor [30], here FGF10 and SHH. This suggests that our study may provide a theoretical framework to describe the development of several other branched organs underlain by similar growth factor/inhibitor couples. Together with other works, this paper finally illustrates that a theoretical approach can be relevant to developmental biology: through combined considerations of genetics, geometry and physics, it can shed light on morphogenesis mechanisms that are hardly intelligible to one discipline alone.

## Supporting Information

Figure S1 **Implementation of the simulations.** Steps of a simulation. Simulations are carried out with Matlab. The growing shape is a polygon with resolution *l_c_* (points are at most spaced by length *l_c_*). In the initial geometry, we compute a mesh for resolution. Then we solve Laplace’s equation on the mesh with finite elements method. These steps are computed with Matlab Partial Differential Equation Toolbox. Then we evaluate the spatial derivative to obtain the gradient. Last, we locally evaluate the obtained gradient for each point of the boundaries to calculate its motion and its new position. If necessary we locally add points so that the resolution remains equal to *l_c_*, and finally obtain the new geometry. Then, we compute a mesh again, etc. Note that the lengths used for this figure were chosen for display purposes and are different from the ones used in the simulations.
(TIFF)

Figure S2 **Parameters and coefficients of the simulations.** (**A**) Equations for the motion of the epithelium and mesothelium. (**B**) Growth functions used in the simulations presented in the paper. *f_lin_* is a linear growth function while *f_sig_* is a sigmoid growth function. (**C**) Table of the values used as parameters for all the simulations presented in the paper. *g* stands for the growth of the mesenchyme, *l_c_* is the numerical resolution of the boundaries (see Fig. S5), and *gs*, *G_0_* and *σ* are parameters of the sigmoid growth response *f_sig_*.
(TIFF)

Figure S3
**Influence of the mesenchyme proliferation term.** (**A**) Occupied space *V_occ_*. *V_occ_* is the space occupied by the lumen over the total space (lumen plus mesenchyme). We plotted *V_occ_* as a function of *g* in the linear case. As one could expect, the occupied space decreases when the mesenchyme proliferation term *g* increases. (**B**) Equilibrium distance. The distance from distal epithelium to mesothelium does not converge during growth, and slowly increases while the whole shape grows. However, the distance rescaled by the external radius of curvature, 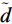, converges towards an equilibrium value 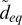. We plotted this rescaled distance at equilibrium as a function of *g* in the linear case, and found that it decreases with *g*. When *g* tends towards zero, the distance tends towards zero. This result suggests that *g* is the relevant parameter to control the equilibrium distance between epithelium and mesothelium.(TIFF)

Figure S4 **Equilibrium distance: Mechanism.** The histograms represent the distribution of the values of the gradient for all points of the epithelium (middle) and mesothelium (bottom), at a late stage of a linear simulation. For the epithelium there are two peaks, one in low gradients (spaces between branches) and one for high gradients (bud tips), which is the one of interest. For the mesothelium we have a normal distribution with only one peak. Reporting these mean gradient values on the growth response curves (top), namely *u_e_* (epithelium, black) and *u_m_* (mesothelium, red), we find that bud tips and mesothelium roughly grow at the same rate (i.e. remain at approximately equal distance). The gradient being a function of local curvature of the boundaries, this suggests that the buds spontaneously adapt their aspect ratio to maintain the gradients such that remains approximately constant.
(TIFF)

Figure S5
**Effective surface tension. (A)** We introduced the length *l_c_* as the spatial resolution of the boundaries. To check the influence of this cut-off length we plotted the mean width of branches λ in the linear case, with *g = 0.5*, as a function of *l_c_*. Error bars represent the standard deviations over the dozens of branches measured. Results show that branches width λ increases linearly with this cut-off. This suggests that in the numerical system, the spatial resolution of the boundaries, *l_c_*, does have the role of a mechanical persistence length, and introduces an effective surface tension, which is physically relevant to the system. The absence of surface tension would lead to infinitely thin branches and to a purely fractal tree. (**B**) Additional simulations show that while branches width λ vary with *l_c_*, the cut-off length has no influence on the equilibrium value of the rescaled distance 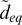 between bud tips and mesothelium. In all other simulations we chose *l_c_ = 0.1.*
(TIFF)

Figure S6 **Additional simulation runs.** Typical simulations with various values of the parameters show that the emergence of a tree is very robust. The inner curves represent successive positions of the epithelium during the run. To keep the figures clear, the mesothelium is plotted only for the end of the simulation. The scale is the same for all simulations, and the values of the parameters are provided Figure S2.
(EPS)

Movie S1 **Movie corresponding to simulation of** **Figure 5A** **.** The color code stands for the gradient of concentration in the mesenchyme (red: high gradients, blue: low gradients). The whole simulation is displayed, with both sides of the initial tube.
(MOV)
