# Supplementary figures and images for: Shape Self-Regulation in Early Lung Morphogenesis
Source: PLoS One. 2012 May 16;7(5):e36925. doi: 10.1371/journal.pone.0036925 (PMC3353953; doi:10.1371/journal.pone.0036925)

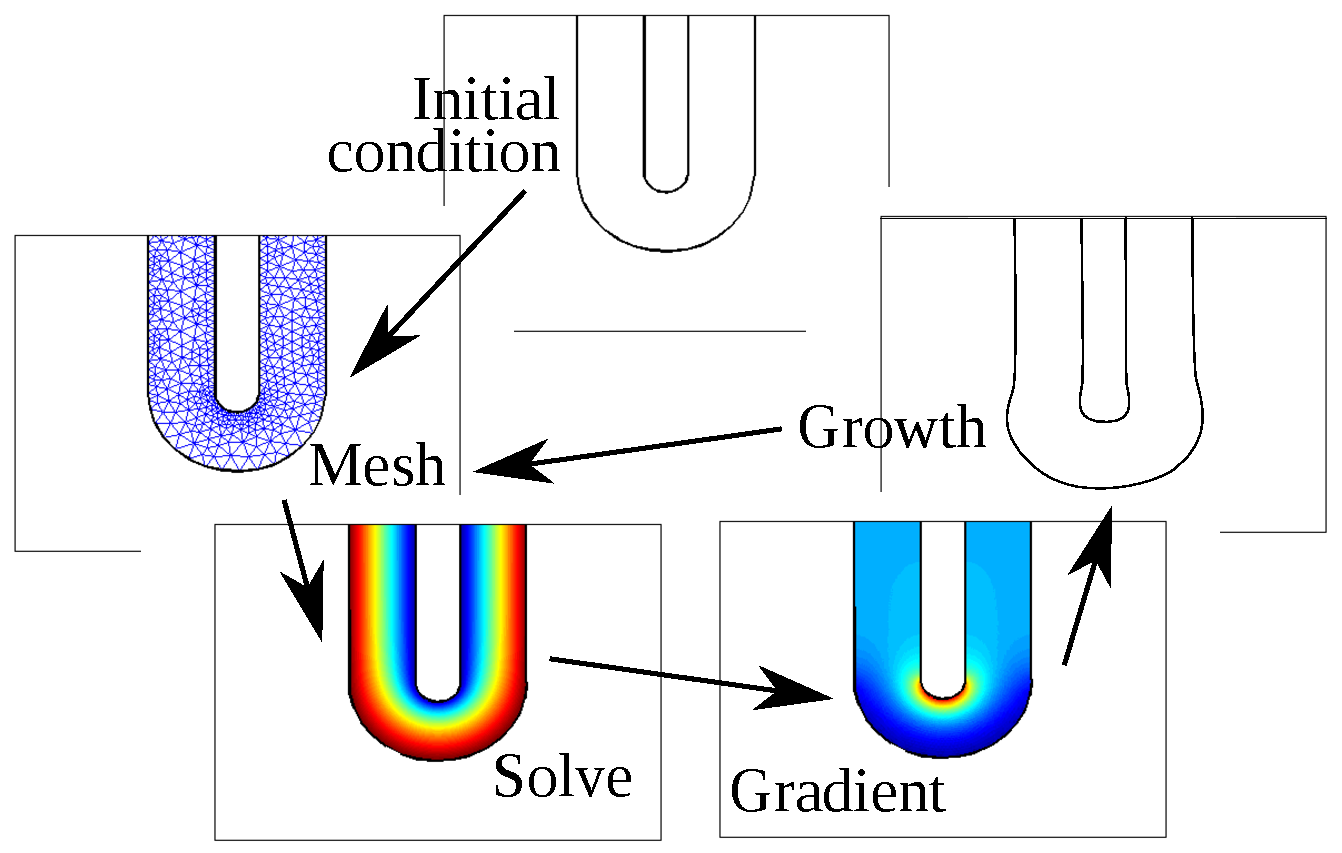

Supplement: Figure S1 — Implementation of the simulations. Steps of a simulation. Simulations are carried out with Matlab. The growing shape is a polygon with resolution lc (points are at most spaced by length lc). In the initial geometry, we compute a mesh for resolution. Then we solve Laplace’s equation on the mesh with finite elements method. These steps are computed with Matlab Partial Differential Equation Toolbox. Then we evaluate the spatial derivative to obtain the gradient. Last, we locally evaluate the obtained gradient for each point of the boundaries to calculate its motion and its new position. If necessary we locally add points so that the resolution remains equal to lc, and finally obtain the new geometry. Then, we compute a mesh again, etc. Note that the lengths used for this figure were chosen for display purposes and are different from the ones used in the simulations. (TIFF) [file pone.0036925.s001.tiff]

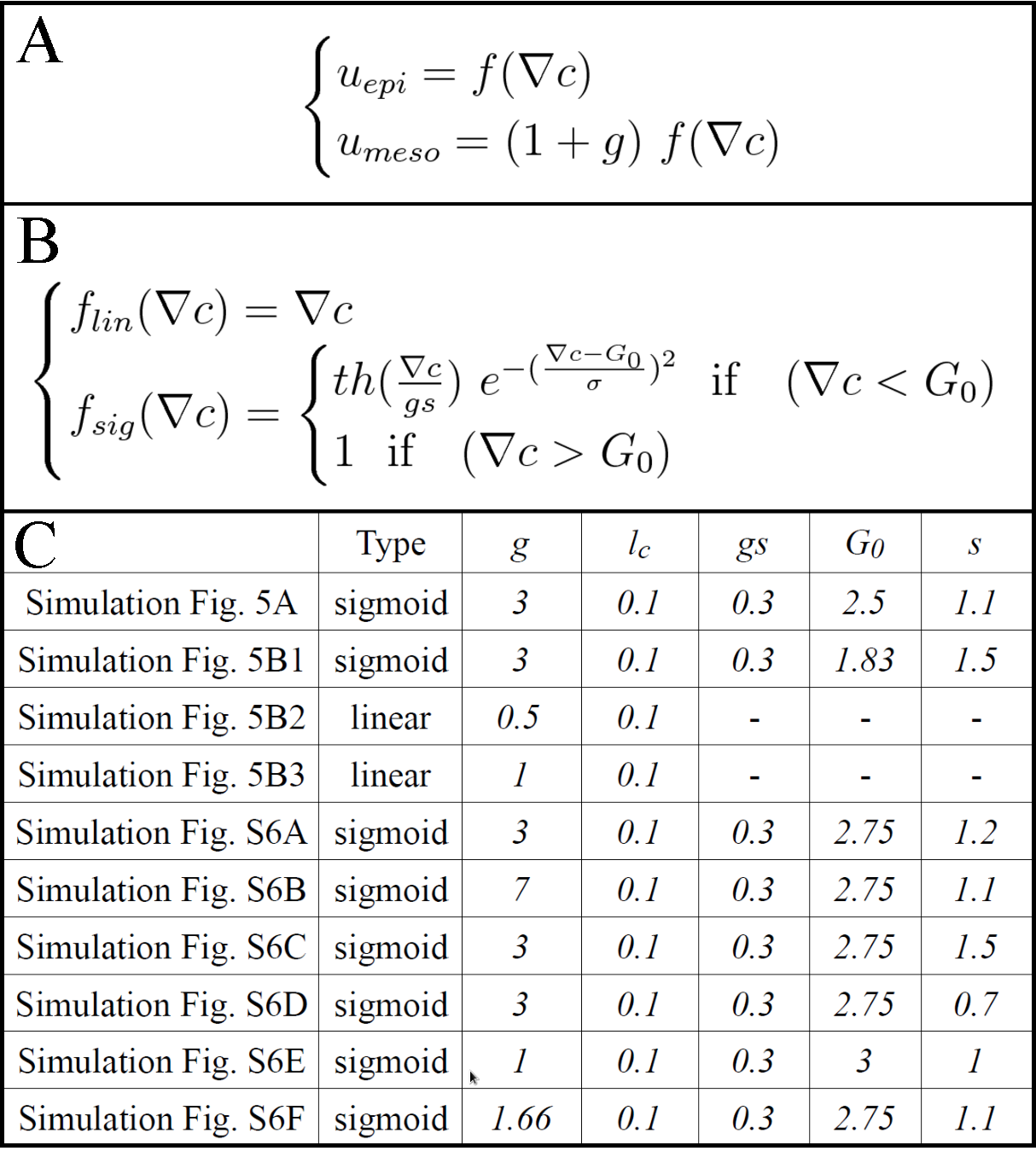

Supplement: Figure S2 — Parameters and coefficients of the simulations. (A) Equations for the motion of the epithelium and mesothelium. (B) Growth functions used in the simulations presented in the paper. flin is a linear growth function while fsig is a sigmoid growth function. (C) Table of the values used as parameters for all the simulations presented in the paper. g stands for the growth of the mesenchyme, lc is the numerical resolution of the boundaries (see Fig. S5), and gs, G0 and σ are parameters of the sigmoid growth response fsig. (TIFF) [file pone.0036925.s002.tiff]

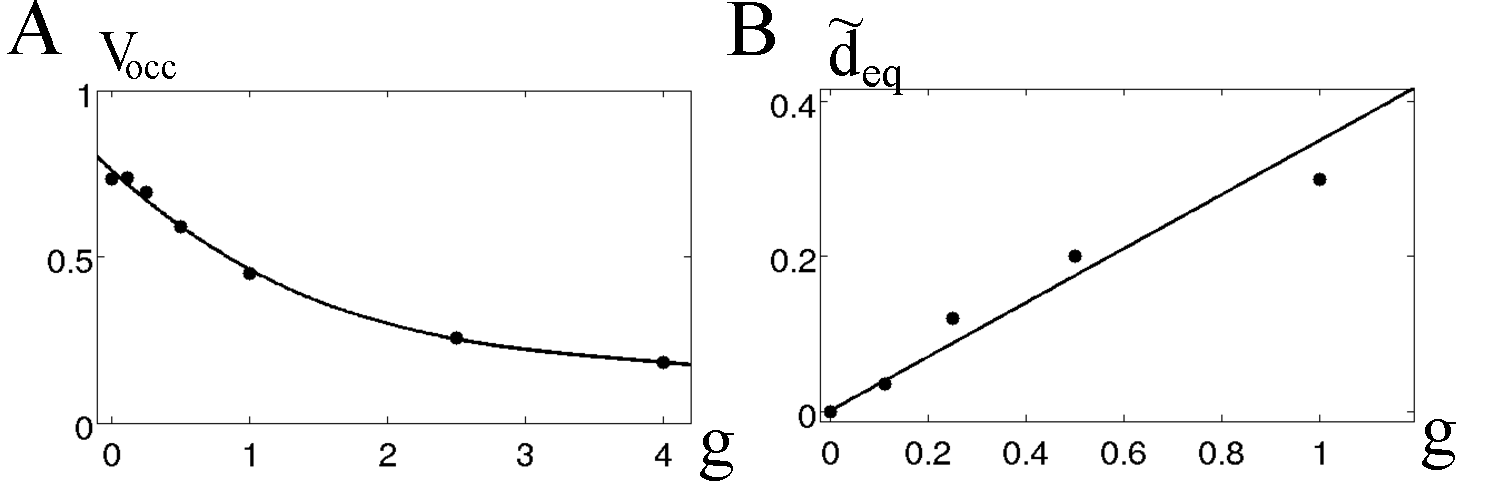

Supplement: Figure S3 — Influence of the mesenchyme proliferation term. (A) Occupied space Vocc. Vocc is the space occupied by the lumen over the total space (lumen plus mesenchyme). We plotted Vocc as a function of g in the linear case. As one could expect, the occupied space decreases when the mesenchyme proliferation term g increases. (B) Equilibrium distance. The distance from distal epithelium to mesothelium does not converge during growth, and slowly increases while the whole shape grows. However, the distance rescaled by the external radius of curvature, , converges towards an equilibrium value . We plotted this rescaled distance at equilibrium as a function of g in the linear case, and found that it decreases with g. When g tends towards zero, the distance tends towards zero. This result suggests that g is the relevant parameter to control the equilibrium distance between epithelium and mesothelium. (TIFF) [file pone.0036925.s003.tiff]

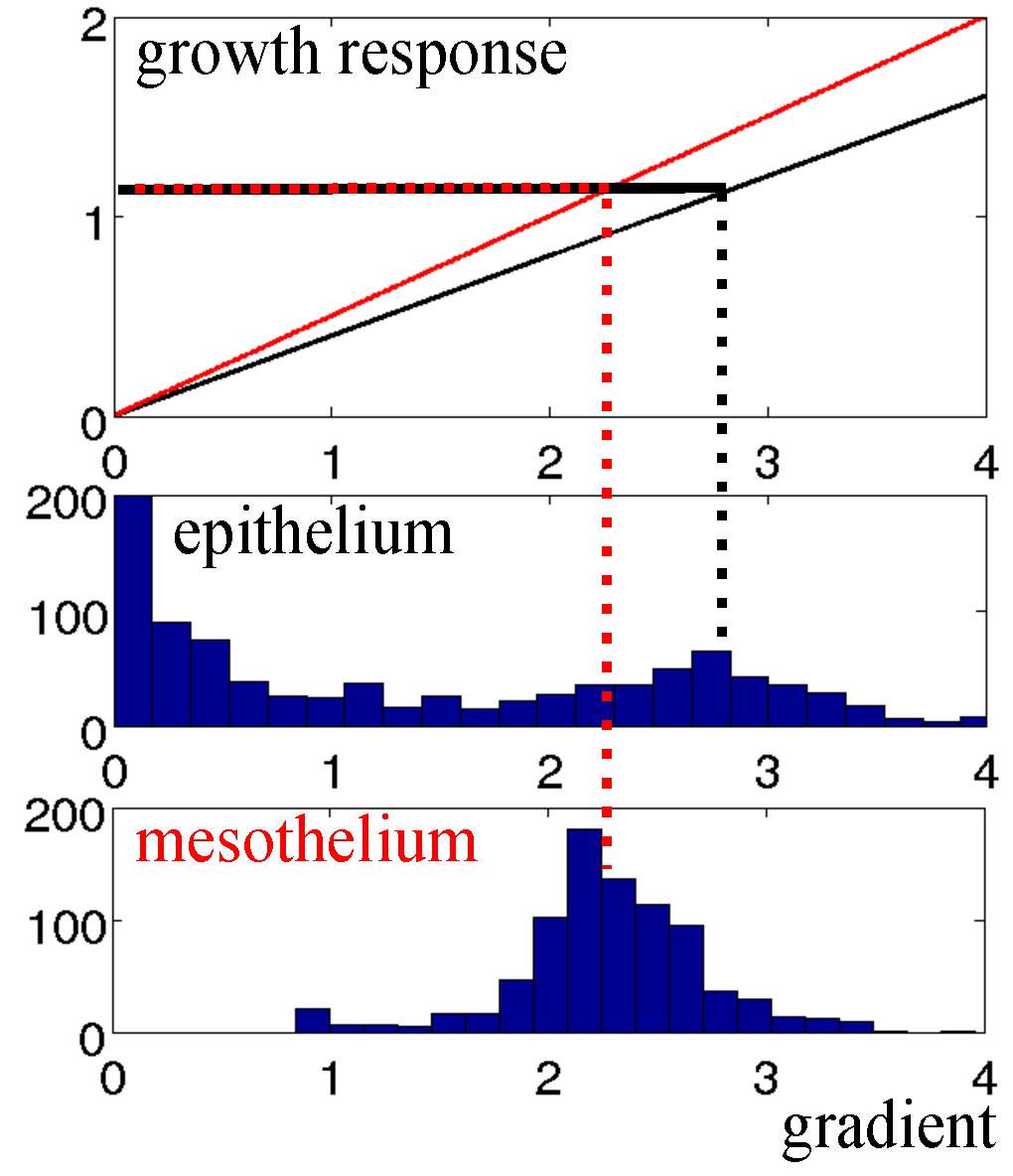

Supplement: Figure S4 — Equilibrium distance: Mechanism. The histograms represent the distribution of the values of the gradient for all points of the epithelium (middle) and mesothelium (bottom), at a late stage of a linear simulation. For the epithelium there are two peaks, one in low gradients (spaces between branches) and one for high gradients (bud tips), which is the one of interest. For the mesothelium we have a normal distribution with only one peak. Reporting these mean gradient values on the growth response curves (top), namely ue (epithelium, black) and um (mesothelium, red), we find that bud tips and mesothelium roughly grow at the same rate (i.e. remain at approximately equal distance). The gradient being a function of local curvature of the boundaries, this suggests that the buds spontaneously adapt their aspect ratio to maintain the gradients such that remains approximately constant. (TIFF) [file pone.0036925.s004.tiff]

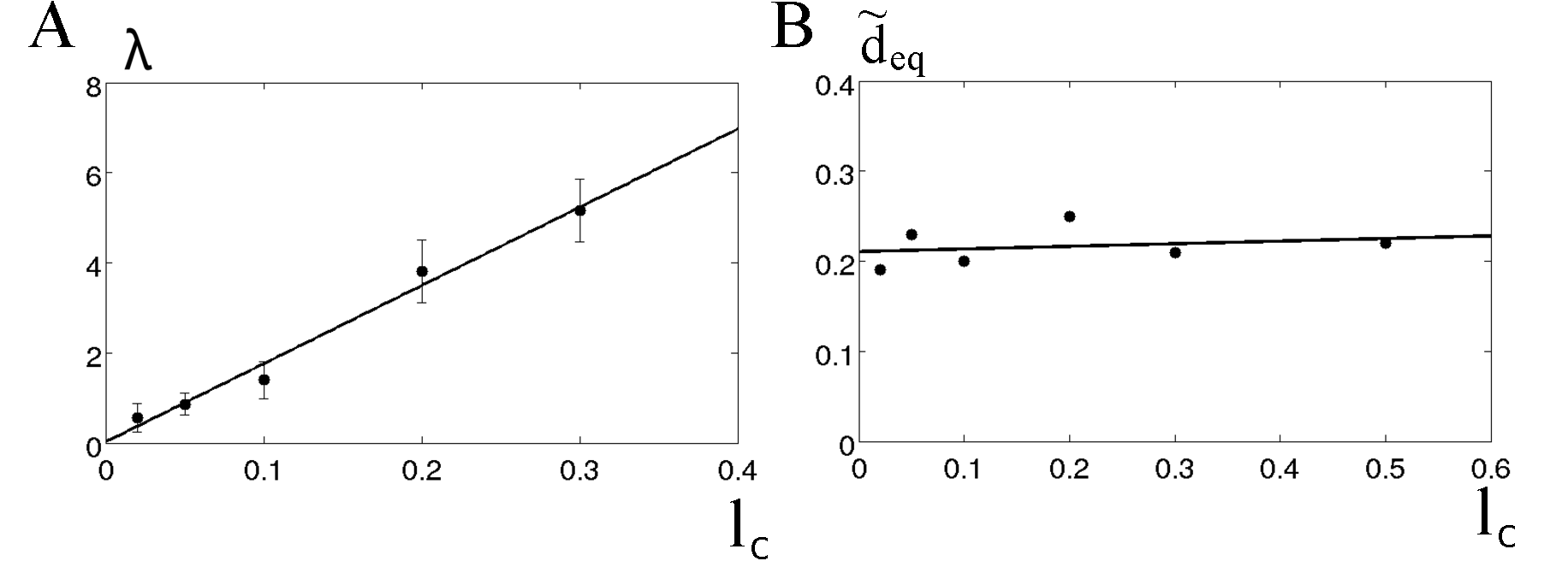

Supplement: Figure S5 — Effective surface tension. (A) We introduced the length lc as the spatial resolution of the boundaries. To check the influence of this cut-off length we plotted the mean width of branches λ in the linear case, with g = 0.5, as a function of lc. Error bars represent the standard deviations over the dozens of branches measured. Results show that branches width λ increases linearly with this cut-off. This suggests that in the numerical system, the spatial resolution of the boundaries, lc, does have the role of a mechanical persistence length, and introduces an effective surface tension, which is physically relevant to the system. The absence of surface tension would lead to infinitely thin branches and to a purely fractal tree. (B) Additional simulations show that while branches width λ vary with lc, the cut-off length has no influence on the equilibrium value of the rescaled distance between bud tips and mesothelium. In all other simulations we chose lc = 0.1. (TIFF) [file pone.0036925.s005.tiff]
